# Electroceutical Treatment of *Pseudomonas aeruginosa* Biofilms

**DOI:** 10.1038/s41598-018-37891-y

**Published:** 2019-02-14

**Authors:** Devendra H. Dusane, Varun Lochab, Travis Jones, Casey W. Peters, Devin Sindeldecker, Amitava Das, Sashwati Roy, Chandan K. Sen, Vish V. Subramaniam, Daniel J. Wozniak, Shaurya Prakash, Paul Stoodley

**Affiliations:** 1 Department of Microbial Infection and Immunity, The Ohio State University, Columbus, Ohio 43210 USA; 2 Department of Mechanical and Aerospace Engineering, The Ohio State University, Columbus, Ohio 43210 USA; 3 Department of Surgery, IU Health Comprehensive Wound Center, Indiana Center for Regenerative Medicine and Engineering, Indiana University School of Medicine, Indianapolis, IN 46202 USA; 4 Comprehensive Wound Center and Department of Surgery, The Ohio State University Wexner Medical Center, Columbus, Ohio 43210 USA; 5 Department of Orthopaedics, The Ohio State University, Columbus, Ohio 43210 USA; 6 National Centre for Advanced Tribology, Mechanical Engineering, University of Southampton, Southampton, UK

## Abstract

Electroceutical wound dressings, especially those involving current flow with silver based electrodes, show promise for treating biofilm infections. However, their mechanism of action is poorly understood. We have developed an *in vitro* agar based model using a bioluminescent strain of *Pseudomonas aeruginosa* to measure loss of activity and killing when direct current was applied. Silver electrodes were overlaid with agar and lawn biofilms grown for 24 h. A 6 V battery with 1 kΩ ballast resistor was used to treat the biofilms for 1 h or 24 h. Loss of bioluminescence and a 4-log reduction in viable cells was achieved over the anode. Scanning electron microscopy showed damaged cells and disrupted biofilm architecture. The antimicrobial activity continued to spread from the anode for at least 2 days, even after turning off the current. Based on possible electrochemical ractions of silver electrodes in chlorine containing medium; pH measurements of the medium post treatment; the time delay between initiation of treatment and observed bactericidal effects; and the presence of chlorotyrosine in the cell lysates, hypochlorous acid is hypothesized to be the chemical agent responsible for the observed (destruction/killing/eradication) of these biofilm forming bacteria. Similar killing was obtained with gels containing only bovine synovial fluid or human serum. These results suggest that our *in vitro* model could serve as a platform for fundamental studies to explore the effects of electrochemical treatment on biofilms, complementing clinical studies with electroceutical dressings.

## Introduction

Biofilms are aggregates of microorganisms with high cell densities embedded in a self-produced extracellular polymeric substance (EPS) that are adherent to each other and/or a surface^1^. Bacteria growing in biofilms cause a wide range of chronic infections^2^ and biofilms in chronic wounds impact over 90% of the infections, which significantly hinders wound healing. Chronic wounds affect over 6.5 million patients with an estimated $25 billion in healthcare costs annually^3^. Standard care approaches of antibiotic treatment and innate immune response are often insufficient for biofilm infection mitigation^4,5^.

**Figure 1:**
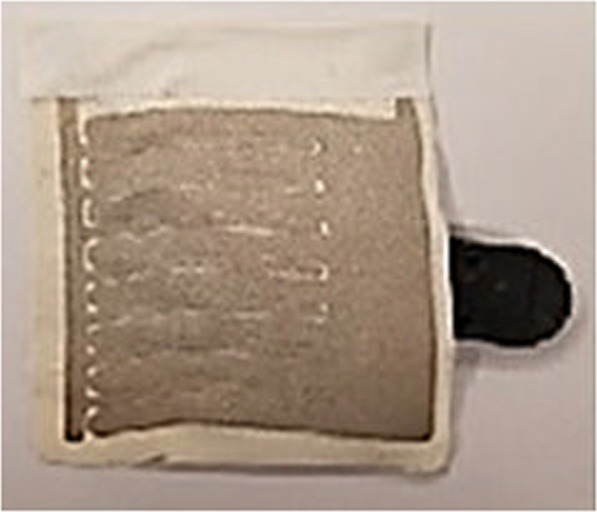
Photograph of an in-house developed prototype of a printed electroceutical dressing (PED) which has Ag/AgCl electrodes printed on silk and powered *via* to a 6 V battery^13^.

While long-term antimicrobial therapy with multiple antibiotics can be effective in some cases^6–8^, treatment failure due to antibiotic resistance^7^, systemic negative effects on the host, and the cost of medical and surgical treatment due to the presence of biofilms are on the rise^9–11^. Development of novel strategies for treatment is necessary, especially given these challenges. Recently, there have been several novel approaches to developing electroceutical dressings, that use either electric fields or currents to remediate biofilms while accelerating wound healing^12–14^. These alternates to antibiotic treatments are in various stages of commercialization but lack a fundamental understanding of the underlying principles behind their efficacy and limitations. The Procellera and Arthrex dressings are examples of electroceuticals that apply electric fields but have no current flow^12^. The patterned electroceutical dressing (PED) is one example (Fig. 1) of the use of direct current (DC) to enhance or replace existing antibiotic regimens^13^. DC has been demonstrated to have killing or eradication efficacy against planktonic bacteria in static and flowing systems^14–18^ with effects dependent on previously used electrode materials such as stainless steel (SS)^19,20^, carbon, platinum, and gold^2,21,22^ and on the composition of the medium^23^.

Costerton and co-workers first reported enhancement of antibiotic efficacy against microbial biofilms by application of current which they termed the “bioelectric effect”^22,24^. For instance, a previous research report showed use of DC at a current density of nearly 2.1 mA/cm^2^ for enhanced elimination of bacterial biofilms in combination with various industrial biocides^14^. In the same work, it was reported that switching the polarity of the flowing current had little or no effect on the survival of *Pseudomonas aeruginosa* (PA) biofilms; however, the combination of direct current and quaternary ammonium biocide significantly increased killing of biofilms grown on SS studs^14^. More recently, hydrogen peroxide produced from an electrochemical scaffold in a liquid bacterial culture medium was used to eradicate PA biofilms^14,25^. In an effort to closely mimic the chemical environment to treat biofilms *in vivo*, Sandvik *et al*.^23^ showed that in an aqueous solution of physiological saline, hypochlorous acid (HOCl) is produced electrochemically and reduced the surface concentration of *Staphylococcus epidermidis* biofilms from approximately 10^7^ to 10^2^ CFU/cm^2^. While most of the previous studies have been done in liquid media that are less applicable to wound biofilms, our current study focuses on the use of an *in vitro* agar based model as it mimics conditions similar to soft tissues in terms of providing a soft surface and a diffusion dominated environment. Mass transfer is an important consideration since in an aqueous environment, mixing of products produced at the electrodes may be rapidly diluted in the bulk fluid such that beneficial effects may only be observed at the electrode. Conversely, in systems with relatively small volumes, products may be produced in sufficiently high concentrations where beneficial effects are observed everywhere in the system. A diffusion dominated environment allows gradients to develop over time, mimicking the environment in tissues.

Wound biofilms usually impact soft tissue *in vivo*, and most *in vitro* electrochemical methods have evaluated interactions of electric current on biofilms in liquid media (e.g. Sandvik 2013). Therefore, a significant knowledge gap remains in how electrical current may impact bacterial biofilms in soft tissue. In the present work, the bioluminescent *P. aeruginosa* Xen41 strain was used to form lawn biofilms on agar plates that serve as *in vitro* platforms to mimic soft tissue. Two rectangular, silver foil electrodes were embedded at the bottom of the agar plate and were not in direct contact with the overlying biofilm. The influence of an applied current on the activity and killing of PA biofilm bacteria was assessed by bioluminescence, viable cell counts, and scanning electron microscope (SEM) imaging.

## Results

### Agar based *in vitro* biofilm model

**Figure 2:**
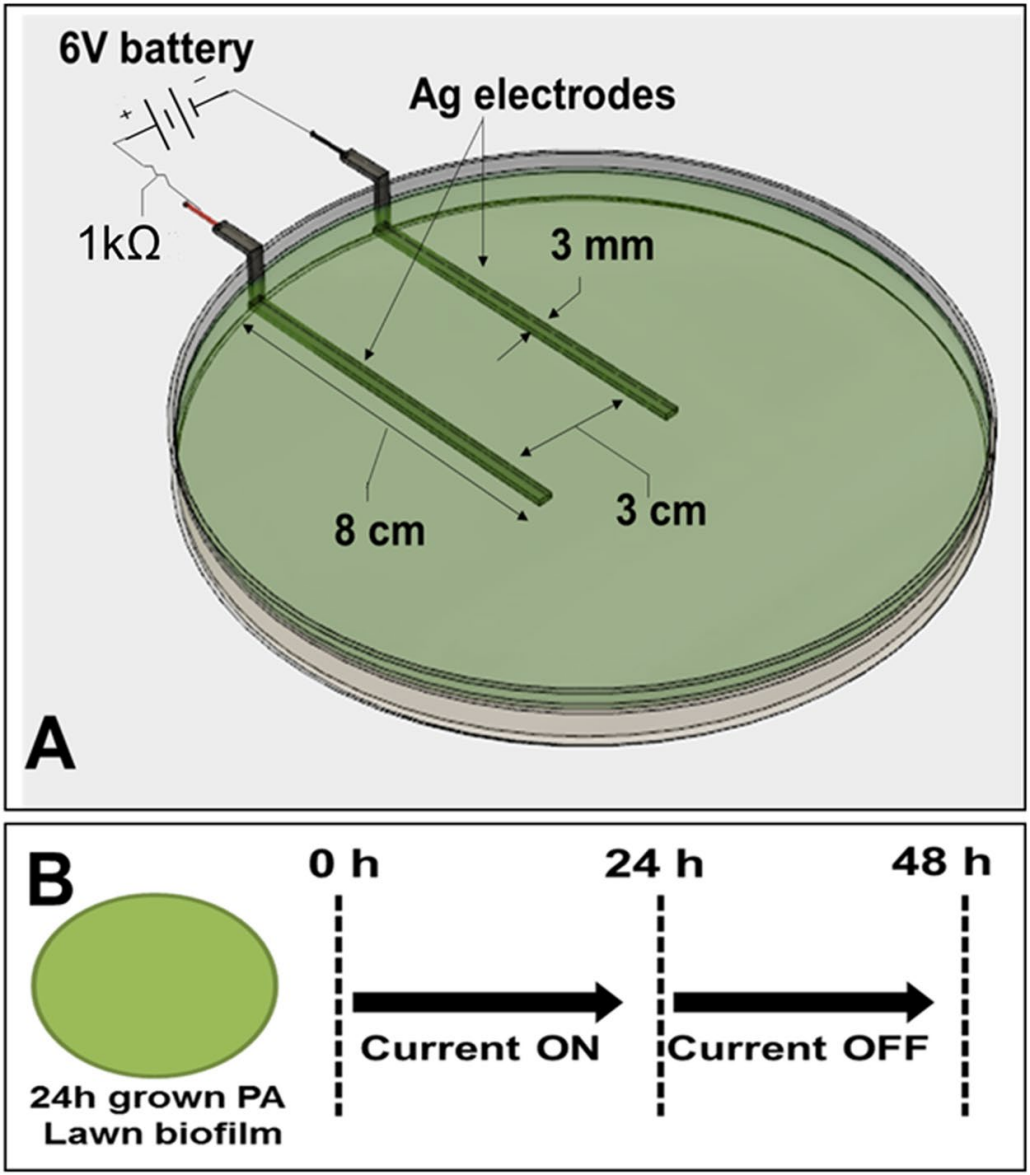
Schematic of *in vitro* agar wound model showing (**A**) Ag electrodes embedded in TSA medium connected to a 6 V battery used to generate current and a 1 kΩ ballast resistor to limit the flow of current and (**B**) Current was applied for either 1 or 24 h and the Petri dishes were incubated upto an additional 24 h after stopping current.

We developed an *in vitro* model for assessing the efficacy of electroceutical treatment on PA biofilms (Fig. 2). After 24 h, the bacteria formed a relatively uniform lawn over the agar. The cell surface density was 4.8 ± 2.4 × 10^9^ CFU/cm^2^ (mean ± 1 SD, n = 3). Bioluminescent intensity was relatively constant over the lawn but diminished over longer periods of time after discontinuing current (i.e. well in excess of 2 days or 48 h), presumably as the bacteria started to become nutrient depleted. The bacterial lawn in this model satisfies the criteria for being considered a biofilm as it is an immobile community of bacteria attached to a living surface (agar) and embedded in an extracellular polymeric matrix (EPS) that they have produced. Moreover, these lawns do not show significant reduction in CFUs at concentrations of the antibiotic tobramycin, which does significantly reduce the planktonic cell count (CFUs) as shown in Fig. S1. Such antibiotic tolerance of these lawns of *P. aeruginosa* is one of the hallmark characteristics of biofilms.

### *P. aeruginosa* biofilms are killed by DC current

**Figure 3:**
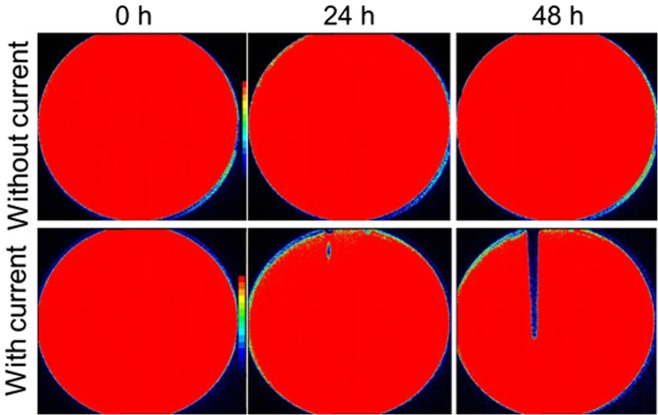
IVIS images showing time dependent killing of PA biofilms after applying current through TSA. Color bars in IVIS images shows red as highly metabolically active/live and blue/black as inactive.

Lawn biofilms of PA-Xen41 grown for 24 h when treated with DC generated from a 6 V source (with 1 kΩ ballast), showed inactivation as observed by the decrease in bioluminescent biofilm bacteria over the anode monitored over a 48 h time window (Figs 3 and S2). Some inactivation at the proximal region of the anode was detected at 24 h with greater inactivation over the whole anode at 48 h. Visual inspection showed that the biofilm had been physically cleared from the anode area as evidenced by a reduction in opacity, presumably reflecting cell lysis in this region (Fig. 3). This was confirmed with *In Vivo* Imaging System (IVIS) imaging and CFU counts.

**Figure 4:**
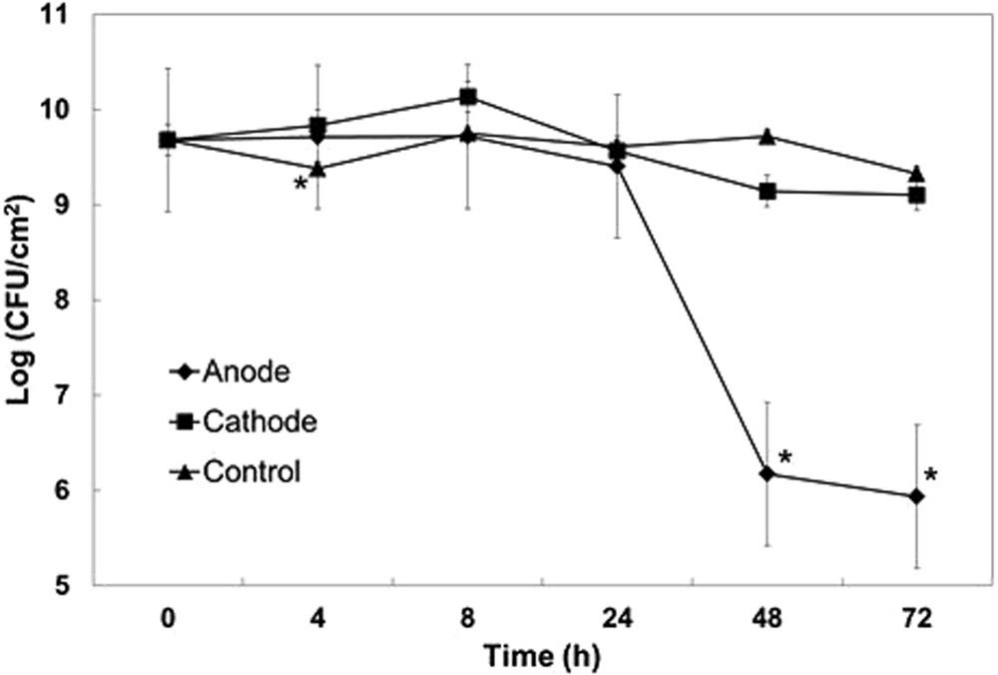
Log CFU of PA biofilms grown on TSA diectly over the anode or cathode. Geometric mean and error bars are ± 1SE (n = 3). Significant differences compared to the control lawn biofilm away from the electrodes are indicated with ‘*’.

**Figure 5:**
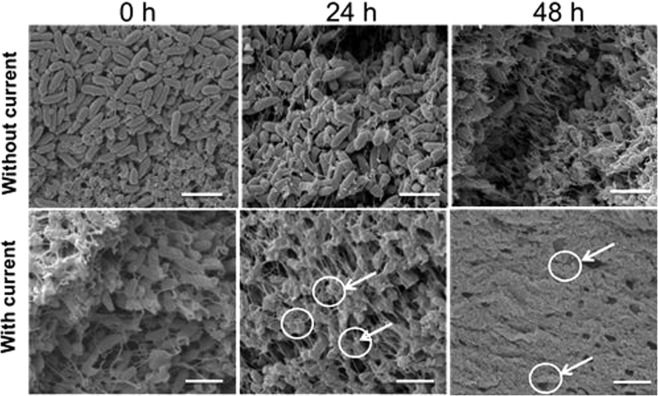
SEM images of electrically untreated control and treated PA biofilms over the anode at different time points. Arrows indicate damaged bacterial cells. Scale bar = 5 µm.

Agar plugs from the treated biofilms over the anode revealed a ~4 log reduction at 48 h and 72 h (p < 0.05 at 48 h and 72 h), whereas there was no statistically significant reduction in cell count at the cathode and electrically untreated control biofilms at these time points (Fig. 4). SEM imaging of sections of the lawn biofilms over the anode depicted healthy rod shaped bacterial cells embedded within the dense biofilms before the current was applied (Fig. 5). However, 24 h post current treatment, it was evident that the cells had begun to lyse. At 48 h, no evidence of distinct individual cells could be found and only bacterial debris were apparent (Fig. 5). In the SEM images of the treated biofilms, there appear to be no healthy individual cells in contrast with the CFU data (Fig. 4), which shows that some viable cells are present. The SEM characterizes a very small region (<50 µm × 50 µm, c.a. 0.0025 mm^2^) directly over the anode, while the punch represents a larger area (12.6 mm^2^) and likely included some live cells at the margin of the killing zone. In this case, the CFU data might actually underestimate the log reduction in the region directly above the anode. Furthermore, since the CFU showed a 4-log reduction, we would expect to see 1 healthy cell out of 10,000, or 1 per average field of view which may be difficult to detect or be buried within the debris of the dead biofilm.

### Silver containing compounds are not responsible for biofilm inhibition

In order to better understand the nature of the electrochemical changes that may be occurring in response to the electric current, EDS analysis was performed across the thickness of the agar over the anode at 48 h (Fig. S3). From the elemental analysis it was evident that Ag is only present in the black layer that forms on the surface of anode and no Ag is detected within the agar or near the biofilm. In contrast, chlorine (Cl) was detected throughout the agar and also in the black layer on the surface of the anode. The atomic percentage of both Ag and Cl is found to be approximately 20% in the black layer that formed on the anode surface and suggests that this black layer acted as a passivating layer of AgCl by stoichiometric proportion. While no Ag is detected above the anode in the agar, killing was observed on the anode. The above results show that an anti-biofilm compound was produced at the anode after applying current in addition to the electrochemical production of AgCl.

### Electric current generates compounds at anode that destroys *P. aeruginosa* biofilms

**Figure 6:**
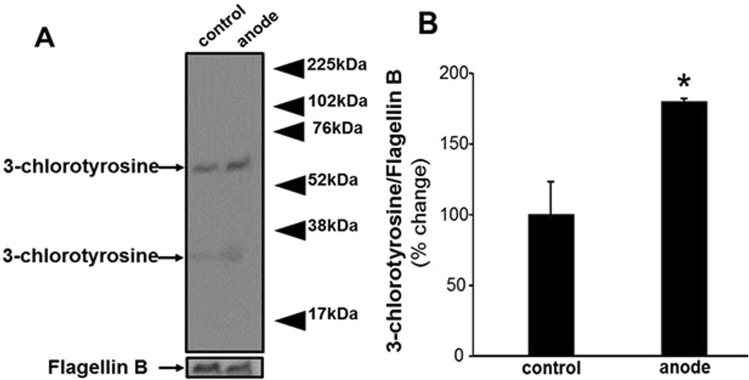
Detection of generation of 3-chlorotyrosine using western blot analysis of PA lawn biofilm treated with current for 24 h. Flagellin B was used as a loading control. (**A**) Representative blot from three independent experiments. (**B**) Densitometry quantification of the western blot normalized to Flagellin B. Data are expressed as mean ± SE (*n* = 3); *P < 0.05 compared to control (no current).

We hypothesize that the cidal agent generated at the anode in our experiments is HOCl. Direct detection of electrochemically produced HOCl is not possible since it reacts with components of the growth media as well^26–28^. Therefore, we assayed for 3-chlorotyrosine, a complex generated by the reaction of HOCl or chloramines with cytoplasmic proteins^29,30^, using western blot analysis of PA lawn biofilms treated with electric current for 24 h. Three independent experiments showed the presence of two bands of chlorotyrosine, as shown in Fig. 6A which displays a representative image. Densitometry quantification of the western blot showed significantly higher content of 3-chlorotyrosine in samples taken at the anode (after exposure to current) as compared to the control (Fig. 6B). The SEM-EDS results along with this chlorotyrosine data serve to further bolster the hypothesis that chlorine chemistry is centrally involved in the observed killing of PA lawn biofilms in this work.

### Growth and killing of *P. aeruginosa* biofilms on agar containing biological fluids

**Figure 7:**
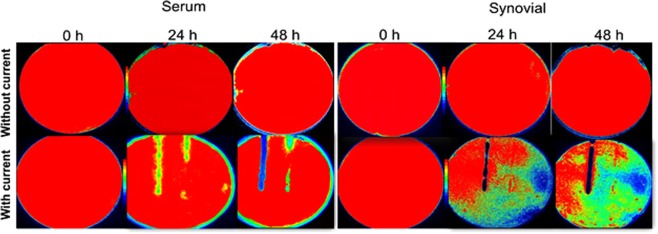
IVIS images showing killing of lawn biofilms of PA-Xen41 grown on agar gels made with human serum or bovine synovial fluid.

**Figure 8:**
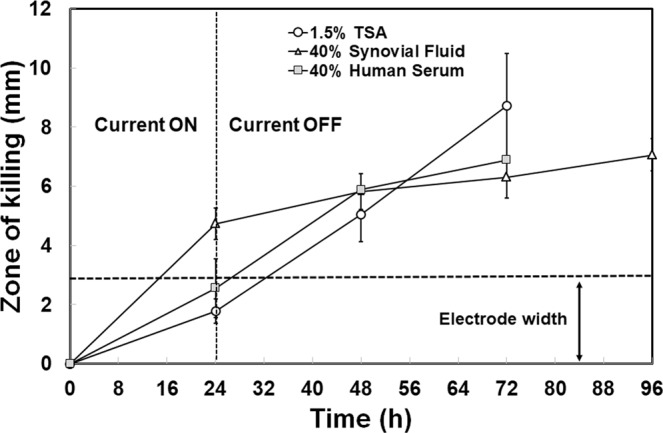
Killing of lawn biofilms of PA grown on either TSA, 40% HS agar or 40% BSF agar after applying current for 24 h. The zone of killing was measured from IVIS images at three equidistant points along the anode.

PA-Xen41 was grown on 1.5% (wt/vol) agar supplemented with either 40% human serum (HS) or 40% bovine synovial fluid (BSF). Compared to the biofilm lawns grown on TSA, relatively greater killing was observed with BSF and HS as can be seen from the reduction in bioluminescence over the anode (Figs 7 and 8). Interestingly, we also observed a reduction in activity over the cathode area with the biofilm lawn on the agar supplemented with HS. Remarkably, the area of the zone of inactivation around the anode continued to increase up to 72 h after the current was turned off (Fig. 8).

### Inhibitory compounds are generated rapidly and are stable over time

**Figure 9:**
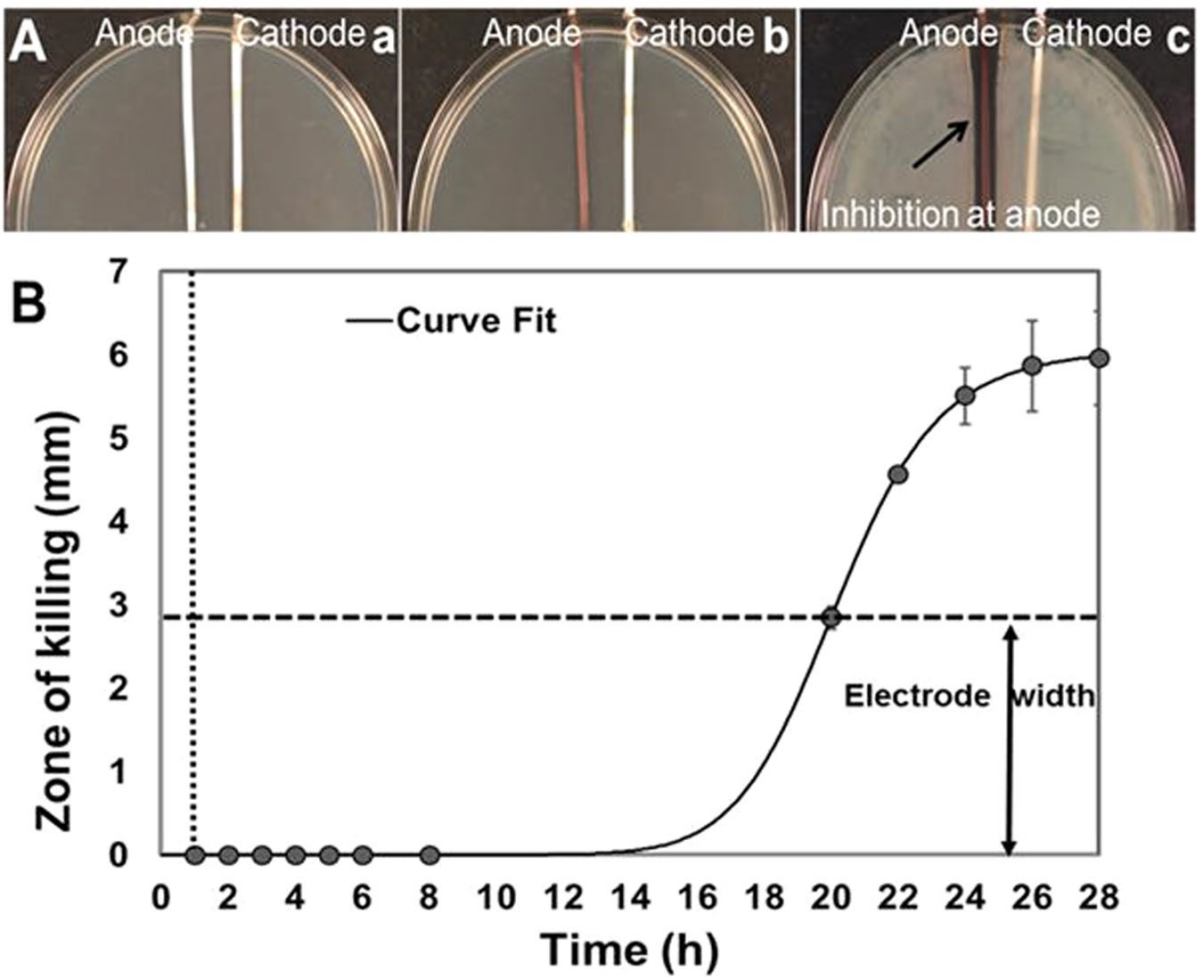
(**A**) Generation of inhibitory product after 1 h of applying current. (a) Petri dishes containing TSA medium with Ag electrodes and without bacteria were treated with DC for 1 h. (b) Current was switched off and the agar spread with PA. (c) After 24 h, a clear zone of inhibition around the anode was evident. (**B**) Killing of lawn biofilms of PA grown on TSA after applying current for 1 h (vertical dashed line). The zone of killing continued to extend beyond the width of the electrodes (indicated by horizontal dashed line) upto 27 h after turning the current off. Vertical dotted line shows the time t = 1 h when current was turned off. Curve fitting was done using the online freeware, MyCurveFit.

To determine the characteristic time scale of generation and stability of the inhibitory compounds, current was driven through the TSA for 1 h at 37 °C and then turned off. A planktonic culture of PA-Xen41 was then spread onto the agar surface and incubated for 24 h at 37 °C. After the incubation period, inhibition of bacterial growth around the anode was visibly evident (Fig. 9A(a–c)). This demonstrates that the antibacterial product was produced at inhibitory concentrations within at most 1 h of electroceutical treatment and was stable well after the current was turned off, when there should be no further generation of electrochemical products.

Similarly, 24 h lawn biofilms (grown prior to electroceutical treatment and subsequently) treated for 1 h with DC, showed a zone of killing that had formed sometime between 7 and 19 h after the current had been turned off (Fig. 9B). As with the observations reported above, the zone of killing around the anode continued to increase over time (Fig. 9B). This observed lag before the biofilm is destroyed is likely due to a combination of the time taken to produce cidal concentrations of the antimicrobial product and the time taken for this cidal substance to diffuse from the anode at the bottom of the agar and culture plate to the lawn biofilm on the agar surface.

### Changes in pH near the electrodes

In order to determine whether current flow causes changes in local pH in the agar, a quantitative measurement of pH was performed using a pH microelectrode (Microelectrodes Inc, USA) over the agar surfaces above the anode, cathode and locations 2 cm laterally away from the electrodes. These measurements at different time points with current flow revealed that the pH is in the range of 6.7 ± 0.1 over the anode, 8.4 ± 0.1 over the cathode and 7.1 ± 0.1 away from the electrodes, confirming the qualitative colorimetric determination (Supplementary Figure S3). It is of note that biofilms are not affected by such variations of pH alone^31,32^.

### Biofilm killing with gold electrodes

Cidal results obtained with Ag electrodes were also observed with Au, and with a larger zone of inhibition over the anode (Fig. S4). However, with Au anodes and cathodes, a zone of inactivation was also observed around the cathode unlike what was observed with Ag electrodes. These differences suggest that electroceutical effects depend on electrode material. The implications of these results are further discussed in the following section.

## Discussion

Electroceutical wound dressings are being developed to treat biofilm related infections^12,33,34^. The class of electroceutical wound dressings that drive a current through the wound contain Ag as at least one of the main functional components, due to cost and due to the previously known antimicrobial properties of Ag^35–37^. A systematic evaluation of the bactericidal effects of electroceutical wound dressing is presently lacking, and this study using an *in vitro* agar model, is a significant advancement in understanding the extensive parametric and mechanistic aspects of why electroceutical dressings are effective in eliminating bacterial biofilms. The PA biofilms used in this work have been demonstrated to be resistant to tobramycin, where corresponding concentrations as high as 16 µg/ml have profound effects in reducing the populations of planktonic bacteria to well below clinical infection thresholds (see Fig. S1). Most previous works have evaluated effects of electrical stimulation of bacteria in liquid media, for example, antibacterial activity has been reportedly observed against *Escherichia coli* in salt solutions^38^; *E. coli*, *Proteus* species and *Klebsiella pneumoniae* in synthetic urine^21^; and *E. coli*, *Staphylococcus aureus* and *Bacillus subtilis* in water^39,40^. In this work, an agar based *in vitro* model relevant for soft tissue wounds has been developed to determine the effect of electrical current on PA biofilms, and is an important step between laboratory and clinical practice.

Using reported wound drainage rates and making reasonable assumptions regarding wound size and wound thickness, the mass transfer characteristics of a typical wound environment were estimated (see Supplemental Information). As described in detail in the Supplementary Data, a typical soft tissue wound was determined to be diffusion limited for HOCl (assuming that it is the active cidal product produced by the electroceutical treatment) with estimates quantified by using the non-dimensional ratio of advection to diffusion over a relevant spatial length scale, i.e., the Peclet number (Pe). Under diffusion limited transport conditions, the transport of compounds such as HOCl generated in the gel after applying current, is controlled by diffusion in the porous medium with little influence from advection. Therefore, any electrochemical reactions generating products at the anode and cathode (and any products resulting from subsequent intermediate reactions with components in the media) will generate gradients from the electrodes and extend into the TSA over length scales determined by chemical from which they were producedspecies-specific diffusivities in a gel or any tissue-like medium, and likely extending from a few millimeters to centimeters. Thus, antimicrobial activity may be apparent at distances from the electrodes over extended periods of time as the generated cidal species continue to diffuse away from the electrodes in the vicinity from which they were produced.

Our work builds on previous work showing the cidal effects of electric current on planktonic bacteria (*P. aeruginosa, S. epidermidis, S. gordonii, E. coli, S. aureus*) in solutions and in bacterial biofilms^11,13,22,37–41^. Where our work differs from these earlier works is in (1) developing a useful *in vitro* agar based platform on which scientific studies may be conducted to determine operating mechanisms for silver-based electroceutical dressings in clinical settings, (2) limiting the current driven through the agar medium, and (3) in determining that the bactericidal effects are due to dissemination of cidal species produced at the electrode. Our agar based *in vitro* model can therefore serve to identify key mechanisms and parameters that need to be optimized for the laboratory-to -clinic translation of electroceutical bactericidal technology.

The key findings of this work on biofilms are summarized in Figs 3–5, where a 4-log reduction in PA biofilms was observed after 48 h of electrical treatment. The rate of reduction in biofilms over 48 h was greater than between 48 h and 72 h. This result may be caused by the possibility that the production of antimicrobial agent at the anode was limited to early time points by passivation AgCl formation as evidenced by the observed black deposit and the EDS data. This suggests that silver as an electrode choice may be self-limiting and might not be best suited for extended treatment periods, since remaining viable cells have the potential to re-establish the infection. However, present clinical practice typically requires dressings to be changed every 3 days so that application of subsequent treatments in intervals of 3 days would reduce or eliminate re-establishment of the infection. Moreover, we anticipate such an electroceutical based technology would be used in conjunction with antibiotic therapy. In future work we will be exploring the use of non-reactive electrode materials such as graphite which will have potential for extended and repeated antimicrobial production. Figures 7 and 8 show the results of PA-Xen41 biofilms grown on gels formed with physiologically relevant fluids such as HS and BSF (both of which contain NaCl at physiological salt concentrations). As can be seen from these figures, PA-Xen41 biofilms were eradicated on agar containing HS and BSF. This bodes well for *in vivo* treatment of such biofilms since chloride ions are known to be present under physiological conditions. Perhaps the most significant result presented in this work is captured in Fig. 9, where the electroceutical treatment is applied for 1 h, then the current shut-off, after which the bacterial lawn was incubated for 24 h. It can be seen from Fig. 9 that cidal effects are evident well after the electric current treatment is off, implying that the observed effect is chemical and diffusional in nature. In other words, a stable antimicrobial agent must have been produced at the anode and subsequently diffused through the agar to later kill the lawn biofilms immediately above the anode.

A reasonable hypothesis from our results as well as from existing work is that the applied current generates an antimicrobial agent or agents at the anode that are stable and able to diffuse to the surface of the agar, whereupon they destroy the biofilm. Sandvik *et al*.^23^ have suggested a possible production of HOCl when current is driven through aqueous media, a potent disinfectant responsible for eradication of biofilms. The experimental results presented in this work cannot unequivocally identify the bactericidal agent(s). Nevertheless, our results do indirectly point to a potential candidate, namely, HOCl. From known electrochemistry of aqueous media containing containing Cl salts^22^, it is possible to identify the following reactions at the anode and cathode:

Anode:

$$Ag(s)+C{l}^{-}\to AgCl(s)+{e}^{-}$$

$$C{l}^{-}+2{H}_{2}O\to {H}_{3}{O}^{+}+HOCl+2{e}^{-}$$

accompanied by

$${H}_{3}{O}^{+}+OC{l}^{-}\rightleftharpoons HOCl+{H}_{2}O$$

where the first two reactions, are surface-mediated and the electrons are injected into the conduction band of the electrode. The third reaction above can proceed in the forward or reverse direction, but is known to progress in the forward direction at pH values below about 7.5^41^. A corresponding reaction at the cathode is:

Cathode

$$2{e}^{-}+2{H}_{2}O\to {H}_{2}+2O{H}^{-}$$

Note that H_3_O^+^ is produced at the anode in the process, while OH^−^ is produced at the cathode. Also noteworthy is the fact that the production of HOCl requires two electrons transferred but only generates a single H_3_O^+^ unlike the reaction at the cathode which produces an OH^−^ for every electron transferred. This is due to the fact that the third reaction at the anode can be driven left or right depending on the pH of the solution. The pKa of HOCl is 7.53, therefore the reaction should favor production of HOCl and not OCl^−^ since the initial pH of the media is ~7.1^41^. This would result in a smaller change in pH at the anode as compared to the change at the cathode. Indeed, this is precisely what was observed in our experiments (Table 1).

**Table 1:** Changes in pH of TSA medium over anode, cathode and locations 2 cm laterally away from the electrodes, measured using a pH microelectrode.

Time (hours)	Anode	Cathode	Away from electrodes
0	7.01 ± 0.1	7.05 ± 0.1	7.08 ± 0.1
24	6.71 ± 0.1	8.40 ± 0.1	7.00 ± 0.1
48	6.79 ± 0.1	8.35 ± 0.1	7.02 ± 0.1

The cathodic and anodic reactions in the electroceutical treatment described in this work indicate localized changes with decrease in pH at the anode and a corresponding increase at the cathode. This is consistent with what has been previously reported in current driven systems^23^ and also with the data reported in the supporting information in this work (Table 1). It is unlikely that the pH changes at the electrodes are responsible for the observed remediation of the PA biofilms since even planktonic PA are known to be unaffected by such excursions in pH on either side of neutral. HOCl is a likely candidate for the observed biofilm inhibition, since biological fluids contain chlorides, speciation of free chlorine is pH dependent, and HOCl is predominantly generated at pH < 7.5 while hypochlorite (OCl^−^) is expected to be produced at pH > 7.5. In addition, HOCl is known to be 100 times more reactive than the hypochlorite ion and a stronger disinfectant, and therefore may explain the bactericidal effects observed at the anode in contrast to the cathode in our experiments^23^.

HOCl can also react with proteins in the media to produce more stable protein chloramines (Fig. 6)^26,42^. Taurine chloramines are produced when the amino acid taurine reacts with HOCl^43^. Such products can be produced from interaction between HOCl and bacteria^26,44^. As shown in Fig. 6, the presence of 3-chlorotyrosine with current along with the observed changes in pH, as well as reported bactericidal effects reported in the literature, bolster the hypothesis that HOCl is present and is likely the cidal agent responsible for the killing of PA biofilms observed in this work.

This work has focused on the use of Ag electrodes in the electroceutical treatment of biofilms. The electrode material plays a significant role in the electrochemical generation of antimicrobial agents. Davis *et al*. (1989) reported the electrode material to be critical in studies applying electric current on bacterial samples in a liquid medium^17^. A gold cathode with carbon and platinum anodes were reported to be the most effective against the urinary tract planktonic pathogens *E. coli* and *P. mirabilis*, while silver, nickel, or copper electrodes corroded to the point of breaking during experiments^17^. In another study, the authors showed application of direct current through platinum electrodes resulted in an increase in the production of reactive oxygen species (ROS) responsible for killing of *S. aureus*, *S. epidermidis* and *P. aeruginosa* biofilms^45^. It is certainly possible that other cidal agents besides HOCl may be active in the PA biofilm remediation observed in our results. In other work, the release of metals from the electrodes has been intentionally used to deliver Ag for treatment^46,47^. It is interesting to note that our EDS analysis at different locations through the agar in our *in vitro* model, showed no presence of Ag beyond the electrode surface (Fig. S3), so that the bactericidal effects of silver alone can be ruled out in our experiments. In the results reported here, both Ag (Fig. 3) and Au (Fig. S4) electrodes were effective in generating electrochemical species leading to eradication of PA biofilms. However, (1) with Au electrodes, killing is observed additionally on the cathode and (2) no chloride layer equivalent to AgCl forms on Au electrodes. Therefore, it is possible that current driven systems using electrodes other than Ag could see excursions in pH well away from neutral and larger than those values measured in this work and potentially involve multiple cidal species.

A drawback of the *in vitro* agar based model introduced in this work is that both voltage and current vary through the course of the electroceutical treatment (Fig. S5), and it is likely that this will occur *in vivo* as well without active real-time sensing and control. Figure S5 shows the voltage measured across the Ag electrodes on the agar side, downstream of the 1 kΩ ballast resistor. In other words, in Fig. S5, $V=(6-1000I)$, where V is in Volts and I is in Amperes. As can be seen from the equation, as the current (I) approaches 0 A, the voltage across the agar approaches 6 V. At the instant current flow begins, it is relatively large (but limited to no more than 6 mA by the ballast), corresponding to the voltage being small across the agar between the electrodes. As the current flows and the electrochemical reactions at the electrode surface and in the agar proceed, the voltage begins to rise (as the AgCl layer forms at the anode and the electrochemical products change the resistance of the agar medium) and the current falls. In Fig. S5, the initial rapid changes in both current and voltage indicate formation of the AgCl layer at the anode, in concert with production of the cidal species that much later in the transient, destroys the biofilm at the agar surface. From known principles of electrochemistry, the magnitude of the voltage at the anode determines which electrochemical reactions are driven (i.e. which species are produced) and the magnitude of the current determines the concentration of species produced. There is therefore a significant opportunity in electroceutical treatments for active control of both current and voltage with real-time sensing of the electrical characteristics of a wound.

Overall, the eradication of biofilms in this study appears to be impacted by a multi-step mechanism, including generation of reactive chlorinated species, changes in pH, and migration of stable reactive species away from the electrodes over extended time periods even after the current has been turned off. Clearly, future work must focus on identifying specific reactive species and migration characteristics to determine efficacy of electrical stimulation *in vivo*. The agar based *in vitro* model presented here can be useful in translation of a laboratory technique to clinical practice. We have identified HOCl as the key cidal agent in electroceuticals based on supporting indirect evidence. HOCl is known as an inorganic bactericidal compound of innate immunity and is effective against a broad range of microorganisms^48–50^. Its presence, however, does raise the potential of cytotoxicity on host cells including immune cells. It is pertinent to emphasize that HOCl is produced by neutrophils and is a natural part of host immune response to pathogens^51^. It is well known that stimulated neutrophils produce HOCl/OCl^−^ at a rate of 12.3 ± 0.8 nmol per 5 × 10^6^ neutrophils (or 2.46 fmol ± 0.2 fmol per stimulated neutrophil)^51^. *In vitro* cytotoxicity profile (L929 cells) and the *in vivo* safety profile of HOCl (at pH 4.0) in various animal models (rabbit and guinea pig) have shown HOCl to be a safe antimicrobial agent with lack of animal toxicity^48^. Minimal bactericidal concentration (MBC) of HOCl against *P. aeruginosa* is reported to be 0.35 µg/mL^48^. With respect to developing the technology as a therapeutic, there is little data on HOCl concentrations and exposure times required to kill *P. aeruginosa*, and whether these can be achieved within tolerable cytotoxicity limits. Sakarya *et al*. (2014) suggested the concentration of HOCl to inactivate *P. aeruginosa* biofilms was 27.5 µg/mL^52^. After 24 h incubation they showed complete killing (a 5 log reduction). However, Chena and Stewart (2000) reported that 15 µg/mL at pH 6.4 only caused a 1-log reduction^53^. Based on these data it appears that the susceptibility is likely system dependent and further *in vitro* and *in vivo* studies are required to determine whether we can operate within a therapeutic window. However, we point out that due to the medical complications in treating chronic biofilm wound infections recent expert opinion suggests early and aggressive treatment^10^, and as such a certain degree of cytotoxicity might outweigh the benefit of controlling the biofilm. HOCl could also be effective in combination with antibiotics where HOCl (at lower than MBC concentrations) could permeabilize the bacterial cells and complete eradication could be possible along with antibiotic treatment. This work lays the foundation for understanding the mechanisms behind the bactericidal effects of electroceutical treatments and can lead to better clinical interventions for antibiotic resistant biofilm infections perhaps even without the need for antibiotics.

## Methods

### Bacterial strain and culture conditions

A bioluminescent strain of *Pseudomonas aeruginosa* (PA) Xen41 (PerkinElmer, USA) was used in all the experiments reported here. PA-Xen41 is a PAO1 luminescent strain that harbors the *luxCDABE* cassette inserted in a constitutively expressed manner^54,55^. The bacterium was cultured in tryptic soy broth (TSB; Sigma Aldrich, USA). The glycerol stock cultures were stored at −80 °C and streaked onto fresh tryptic soy agar (TSA) plates containing 1.5% agar in TSB, that were incubated for 24 h at 37 °C in 5% CO_2_. The isolated colonies from the TSA plate were then transferred to 20 mL of TSB and incubated overnight on an incubator shaker set at a temperature of 37 °C and speed of 200 rpm.

### Agar based wound model for *in vitro* electroceutical testing

Silver electrodes were cut from 0.1 mm thick, 99.9% pure Ag foil (Sigma-Aldrich, USA). All electrodes were 3 mm wide and 10 cm in length. A pair of electrodes was laid flat at a distance 3 cm apart in a 150 mm diameter polystyrene Petri dish (Fisher Scientific, USA) with both electrodes extending 8 cm from the walls of the dish. The remaining 2 cm length of the Ag electrodes extended outside the Petri dish and were connected to a 6 V battery pack. The battery pack comprised two 3 V batteries (CR2032, Energizer, USA) in series with a switch and a 1kΩ ballast resistor (Fig. S2) to limit the total current to below 6 mA when the switch is turned on (Fig. 2). TSA (55 mL) was poured on the flattened electrodes placed in the Petri dishes. The agar was allowed to solidify to room temperature. The measured current was observed to decrease from 3.5 ± 0.38 mA (where ± indicates range of two independent observations) at time t = 0 when the switch was turned on, to 0.15 ± 0.06 mA (150 μA ± 60 μA) over a period of 24 h and therefore all experiments were limited to no more than 24 h current passage (Fig. S5).

### Preparing lawn biofilms of *P. aeruginosa*

Lawn biofilms of PA-Xen 41 were generated by spreading the overnight culture on TSA with the Ag electrodes embedded underneath the agar layer, which is approximately 3.6 mm thick. Briefly, 100 µL of the overnight PA-Xen41 culture grown in TSB was mixed with 9.9 mL TSB to make a 1:100 dilution. 400 µL of the diluted culture was spread onto the TSA with embedded electrodes contained in a polystyrene petri-dish (150 mm × 15 mm, Fisher Scientific, USA). The petri-dishes were incubated at 37 °C in 5% CO_2_ for 24 h to develop lawn biofilms of PA-Xen41. The PA lawns were verified as being biofilms by measuring their response to the antibiotic, tobramyacin (Fig. S1).

### Killing of *P. aeruginosa* biofilms by electric current

The bioluminescent strain of PA-Xen41 was used in the data reported here since it enables easy monitoring of growth and metabolic activity based upon changes in intensity^56^, where red indicates active cells and blue/black represents less active or dead cells (Fig. 3; IVIS images). Cell death of course is confirmed by CFU counts. The lawn biofilms of PA-Xen41 formed for 24 h on the TSA surface were subjected to electroceutical treatment by driving electric current through the embedded Ag electrodes for 24 h. As described earlier, a 6 V battery pack with a 1 kΩ ballast resistor connected in series was used to drive the current. The Petri dishes were kept at 37 °C for 24 h in 5% CO_2_ after which the battery was disconnected and the petri-dishes were incubated for an additional 24 h. Control samples were lawn biofilms formed for 24 h and incubated further for 48 h without electroceutical treatment.

### Colony forming units of lawn biofilms at different time intervals

The colony forming units (CFU/cm^2^) of lawn biofilms were measured (i) before applying current at time t = 0 h, (ii) when current was stopped at 24 h, and (iii) after incubation without current till the 48 h time point. Agar cylindrical plugs (4 mm in diameter) from different areas of the plates (over anode and cathode) were removed using biopsy punches with the plunger system (Integra Miltex, Fisher Scientific, USA). The agar plugs were placed in sterile PBS, vortexed, serially diluted and plated onto TSA medium. The petri-dishes were incubated for 24 h at 37 °C and colonies were enumerated. Control samples were lawn biofilms formed for 24 h and incubated further for 48 h without electroceutical treatment.

### Scanning electron microscope (SEM) imaging

Lawn biofilms of PA-Xen41 grown on TSA medium were imaged using a FEI Nova Nano SEM 400 (FEI™, Hillsboro, OR) with a field-emission gun electron source, at an accelerating voltage of 5 kV. Biopsy punch samples (~4 mm diameter) were collected from the anode region at different time points (0, 24, and 48 h). The punched out agar blocks with the biofilms were then placed in 24 well microtiter plates and the biofilm was fixed in a glutaraldehyde buffer for 48 h at 4 °C followed by dehydration with graded ethanol. The samples were then chemically dried overnight with hexamethyldisilazane (HMDS, Ted Pella Inc.). Before imaging, the samples were mounted on an aluminum stub and sputter coated with Au-palladium to minimize sample charging when exposed to the electron beam^57–59^.

### Energy dispersive x-ray spectroscopic (EDS) analysis

EDS elemental analysis was performed to determine the abundance of the elements contained in the inhibitory compound produced after applying current and to determine whether or not Ag leaches out from the electrodes into the agar. EDS analysis was performed using a Hitachi S-3000H SEM with EDAX Falcon EDS detection system model 132–10. The agar punches were obtained as mentioned above in the section on SEM analysis, and the samples were sliced (Fig. S2) to obtain depth profiles of elemental composition. The images of the EDS sample were taken without any coating, and using the SEM operating at 5 kV equipped with an Everhart-Thornley secondary electron detector.

### Detection of chemical nature of bactericidal compound produced by electroceutical process using western blot analysis

To investigate our hypothesis that HOCl may be a key agent in the observed killing of PA biofilms, we performed western blot analysis using a primary antibody (Hycult Biotech Inc., PA) which recognizes 3-chlorotyrosine protein adducts formed when HOCl reacts with proteins^27,60–62^. Briefly, the PA lawn biofilms were formed on TSA and electroceutical treatment was applied for 24 h. The biofilm over the anode was scraped off using sterile disposable inoculating loop (Fisherbrand, USA) and collected in a sterile eppendorf tube containing 1 mL of PBS and labelled (as anode). The control eppendorf tube contained 1 mL PBS with scraped 24 h lawn biofilm of PA from TSA (without electroceutical treatment). Both the eppendorf tubes were centrifuged at 10,000 rpm for 5 min. The supernatant was discarded and the resulting pellet was used for analysis. After labelling with the primary antibody, the signals were visualized using corresponding HRP-conjugated secondary antibody (1: 2,000; GE Healthcare Life Sciences, PA) and ECL Plus™ Western Blotting Detection Reagents (GE Healthcare Life Sciences, PA). Membranes were stripped and re-probed with anti-Flagellin B which served as the loading control^28^.

### Killing of lawn biofilms grown on agar surface with biological fluids

To determine whether or not biological fluids affect the killing of PA lawn biofilms using electric current, two additional sets of agar media were prepared using human serum (HS, Type AB, Atlanta Biologicals, GA) and bovine synovial fluid (BSF, Lampire Biological Laboratories, USA). HS or BSF containing agar was prepared with the biological fluid (400 mL/L) in distilled water and the solidifying agent (agar, 1.5% wt./vol.). The biological fluids were added to pre-sterilized media cooled to 40 °C to avoid degradation or coagulation of proteins and to preserve the constituents of the fluids. PA-Xen41 grown overnight was diluted (1:100 in TSB) and 400 µL of the diluted culture was spread onto the respective media containing Ag foils, as already described above for TSA media. Direct current was applied to the pre-grown lawn biofilms for 24 h. The battery was removed at 24 h and the petri-dishes were incubated further for another 24 h. Images (white light and IVIS) were taken at t = 0 (when current was turned on), 24 (current off), and 48 h (24 h after the current was turned off), respectively. Zone of inhibition measurements were performed on IVIS images. The widths of inhibition zones at distances of 2 cm (from electrode entering the agar), 4 cm (midway) and 6 cm (towards tip of the anode) beginning from top of the anode, were measured and the average width of the three measurements was plotted at each time point.

### Effect of varying current on killing of *P. aeruginosa* Xen41 biofilms

We anticipated that the magnitude of the current could impact the concentration of cidal specie(s) electrochemically produced and affect the extent of killing of the biofilm. In order to explore this possible effect, different ballast resistances were used and the killing efficiency quantified using IVIS imaging. With the different ballast resistors (6 kΩ, 1 kΩ and 500Ω) in series with the 6 V battery pack, initial currents were measured to be 0.65 ± 0.01 mA (650 ± 10 μA), 3.50 ± 0.38 mA (3500 ± 380 μA), and 5.85 ± 0.08 mA (5850 ± 80 μA) respectively (mean ± 1 SD, n = 2). With current flowing for 24 h, the final current recorded was 0.09 ± 0.002 mA (90 ± 2 μA), 0.15 ± 0.06 mA (150 ± 60 μA), and 0.157 ± 0.007 mA (157 ± 7 μA) respectively. Although qualitatively higher eradication of the biofilm was observable at 500Ω (Fig. S2), visible changes were also observed in the agar. In contrast, killing with the 1 kΩ ballast was relatively greater than with the 6 kΩ ballast with no degradation or visible change in the agar. Consequently, the 1 kΩ ballast resistor connected in series with the 6 V batteries was used in all the experiments reported here.

### Effect of short term electroceutical treatment on generation of bactericidal compound

Ag electrodes were embedded under TSA in 150 mm Petri dishes and current was applied for 1 h using 6 V batteries connected in series with 1 kΩ ballast. The current was then turned off after 1 h, and 12 h grown culture of PA-Xen41 (diluted 1:100) was spread on the surface of TSA. The Petri dishes were incubated for 24 h at 37 °C with 5% CO_2_.

### Changes in pH of the agar medium in the presence of direct current

To monitor changes in pH, the microelectrode (MI-710, Microelectrodes, Inc, NH, USA) was used. Direct current was applied as shown in Fig. 2, using 6 V with a 1 kΩ ballast. The pH was measured over the anode, cathode and different locations 2 cm laterally away from the electrodes by placing the microelectrode on the surface of agar. The pH of TSA medium was measured before (t = 0) and after (t = 24, 48 h) applied current.

### Effect of electrode material on killing of *P. aeruginosa* Xen41 biofilms

In order to determine whether the electrode material has any influence on killing of PA biofilms, Ag electrodes were replaced by Au (99.987% pure, Alfa Aesar) electrodes. The TSA medium was poured over the Au foils in Petri dishes, allowed to gel and lawn biofilms were then grown on top for 24 h, as described previously. Current was driven through the Au electrodes for 24 h. Plates were incubated for a further 48 h (24 h without DC, as with all Ag foil electrode experiments) and IVIS images were taken at that time.

### Statistical analysis

All experiments were performed in triplicates, unless otherwise indicated for specific experiments. Control and treated samples were compared by paired, two test distribution using Student’s *t*-test where *P* < 0.05 was considered a statistically significant difference. Data represented in bar graphs are plotted as the mean ± standard error (SE) from the mean.

## Supplementary information

Supplementary information

## References

[1] Flemming H-C, Wingender J, Szewzyk U, Steinberg P, Rice A (2016). S. Biofilms: an emergent form of bacterial life. Nature Reviews Microbiology.

[2] del Pozo JL, Patel R (2007). The challenge of treating biofilm-associated bacterial infections. Clinical pharmacology and therapeutics.

[3] Sen CK (2009). Human skin wounds: a major and snowballing threat to public health and the economy. Wound Repair and Regeneration.

[4] Wu H, Moser C, Wang H-Z, Høiby N, Song Z-J (2015). Strategies for combating bacterial biofilm infections. International journal of oral science.

[5] Koo, H., Allan, R. N., Howlin, R. P., Stoodley, P. & Hall-Stoodley, L. Targeting microbial biofilms: current and prospective therapeutic strategies. *Nature reviews. Microbiology* (2017). 10.1038/nrmicro.2017.99 PMC5685531 28944770

[6] Konig DP, Schierholz JM, Munnich U, Rutt J (2001). Treatment of staphylococcal implant infection with rifampicin-ciprofloxacin in stable implants. Archives of orthopaedic and trauma surgery.

[7] Pavoni GL (2004). Conservative medical therapy of prosthetic joint infections: retrospective analysis of an 8-year experience. Clinical microbiology and infection: the official publication of the European Society of Clinical Microbiology and Infectious Diseases.

[8] Zimmerli W, Widmer AF, Blatter M, Frei R, Ochsner PE (1998). Role of rifampin for treatment of orthopedic implant-related staphylococcal infections: a randomized controlled trial. Foreign-Body Infection (FBI) Study Group. Jama.

[9] Darouiche RO (2004). Treatment of infections associated with surgical implants. The New England journal of medicine.

[10] Schultz, G. *et al*. Consensus guidelines for the identification and treatment of biofilms in chronic non-healing wounds. *Wound repair and regeneration: official publication of the Wound Healing Society and the European Tissue Repair Society*, 10.1111/wrr.12590 (2017). 10.1111/wrr.12590 28960634

[11] Snyder RJ (2017). Wound Biofilm: Current Perspectives and Strategies on Biofilm Disruption and Treatments. Wounds: a compendium of clinical research and practice.

[12] Banerjee J (2015). Silver-zinc redox-coupled electroceutical wound dressing disrupts bacterial biofilm. PloS one.

[13] Prakash, S. *et al*. *Antimicrobial wound dressing*. United States patent (2016).

[14] Sultana ST, Babauta JT, Beyenal H (2015). Electrochemical biofilm control: a review. Biofouling.

[15] Istanbullu O, Babauta J, Duc Nguyen H, Beyenal H (2012). Electrochemical biofilm control: mechanism of action. Biofouling.

[16] Davis CP, Arnett D, Warren MM (1982). Iontophoretic killing of Escherichia coli in static fluid and in a model catheter system. Journal of clinical microbiology.

[17] Davis CP, Weinberg S, Anderson MD, Rao GM, Warren MM (1989). Effects of microamperage, medium, and bacterial concentration on iontophoretic killing of bacteria in fluid. Antimicrobial agents and chemotherapy.

[18] Davis CP, Wagle N, Anderson MD, Warren MM (1992). Iontophoresis generates an antimicrobial effect that remains after iontophoresis ceases. Antimicrobial agents and chemotherapy.

[19] Wattanakaroon W, Stewart PS (2000). Electrical enhancement of Streptococcus gordonii biofilm killing by gentamicin. Archives of Oral Biology.

[20] Caubet R (2004). A radio frequency electric current enhances antibiotic efficacy against bacterial biofilms. Antimicrobial agents and chemotherapy.

[21] Davis CP, Wagle N, Anderson MD, Warren MM (1991). Bacterial and fungal killing by iontophoresis with long-lived electrodes. Antimicrobial agents and chemotherapy.

[22] Khoury AE, Lam K, Ellis B, Costerton JW (1992). Prevention and control of bacterial infections associated with medical devices. ASAIO journal (American Society for Artificial Internal Organs: 1992).

[23] Sandvik EL, McLeod BR, Parker AE, Stewart PS (2013). Direct electric current treatment under physiologic saline conditions kills Staphylococcus epidermidis biofilms via electrolytic generation of hypochlorous acid. PloS one.

[24] Blenkinsopp SA, Khoury AE, Costerton JW (1992). Electrical enhancement of biocide efficacy against Pseudomonas aeruginosa biofilms. Applied and environmental microbiology.

[25] Sultana ST, Call DR, Beyenal H (2016). Eradication of Pseudomonas aeruginosa biofilms and persister cells using an electrochemical scaffold and enhanced antibiotic susceptibility. NPJ biofilms and microbiomes.

[26] Pattison DI, Hawkins CL, Davies MJ (2007). Hypochlorous acid-mediated protein oxidation: how important are chloramine transfer reactions and protein tertiary structure?. Biochemistry.

[27] Roy S (2007). P21waf1/cip1/sdi1 as a central regulator of inducible smooth muscle actin expression and differentiation of cardiac fibroblasts to myofibroblasts. Molecular biology of the cell.

[28] Barki, K. G. *et al*. Electric Field Based Dressing Disrupts Mixed-Species Bacterial Biofilm Infection and Restores Functional Wound Healing. *Ann Surg*, 10.1097/sla.0000000000002504 (2017). 10.1097/SLA.0000000000002504 PMC6568008 29099398

[29] Drabik G, Naskalski JW (2001). Chlorination of N-acetyltyrosine with HOCl, chloramines, and myeloperoxidase-hydrogen peroxide-chloride system. Acta biochimica Polonica.

[30] Domigan NM, Charlton TS, Duncan MW, Winterbourn CC, Kettle AJ (1995). Chlorination of tyrosyl residues in peptides by myeloperoxidase and human neutrophils. The Journal of biological chemistry.

[31] Xiong YQ, Caillon J, Drugeon H, Potel G, Baron D (1996). Influence of pH on adaptive resistance of Pseudomonas aeruginosa to aminoglycosides and their postantibiotic effects. Antimicrobial agents and chemotherapy.

[32] Jones EM, Cochrane CA, Percival SL (2015). The Effect of pH on the Extracellular Matrix and Biofilms. Advances in Wound Care.

[33] Ganesh K (2015). A Wireless Electroceutical Wound Dressing Disrupts Mixed Species Bacterial Biofilm In A Porcine Preclinical Model. Wound Repair and Regeneration.

[34] Doxsee, K., Berthelot, R. & Neethirajan, S. Electroceutical disinfection strategies impair the motility of pathogenic Pseudomonas aeruginosa and Escherichia coli. *bioRxiv*, 088120 (2016).

[35] Percival SL, Bowler PG, Dolman J (2007). Antimicrobial activity of silver-containing dressings on wound microorganisms using an *in vitro* biofilm model. International wound journal.

[36] O’Neill MA (2003). Antimicrobial properties of silver-containing wound dressings: a microcalorimetric study. International journal of pharmaceutics.

[37] Brett DW (2006). A discussion of silver as an antimicrobial agent: alleviating the confusion. Ostomy/wound management.

[38] Pareilleux A, Sicard N (1970). Lethal Effects of Electric Current on Escherichia coli. Applied Microbiology.

[39] Matsunaga T, Nakasono S, Masuda S (1992). Electrochemical sterilization of bacteria absorbed on granular activated carbon. FEMS microbiology letters.

[40] Matsunaga T (1992). Disinfection of drinking water by using a novel electrochemical reactor employing carbon-cloth electrodes. Applied and environmental microbiology.

[41] Horváth AK, Nagypál I (2000). Kinetics and mechanism of the reaction between hypochlorous acid and tetrathionate ion. International Journal of Chemical Kinetics.

[42] Pattison DI, Davies MJ (2001). Absolute rate constants for the reaction of hypochlorous acid with protein side chains and peptide bonds. Chemical research in toxicology.

[43] De Carvalho Bertozo L, Morgon NH, De Souza AR, Ximenes VF (2016). Taurine bromamine: reactivity of an endogenous and exogenous anti-inflammatory and antimicrobial amino acid derivative. Biomolecules.

[44] Gray MJ, Wholey W-Y, Jakob U (2013). Bacterial responses to reactive chlorine species. Annual review of microbiology.

[45] Brinkman CL (2016). Exposure of Bacterial Biofilms to Electrical Current Leads to Cell Death Mediated in Part by Reactive Oxygen Species. PloS one.

[46] Berger T, Spadaro J, Bierman R, Chapin S, Becker R (1976). Antifungal properties of electrically generated metallic ions. Antimicrobial agents and chemotherapy.

[47] Berger T, Spadaro J, Chapin S, Becker R (1976). Electrically generated silver ions: quantitative effects on bacterial and mammalian cells. Antimicrobial agents and chemotherapy.

[48] Wang, L. *et al*. Hypochlorous acid as a potential wound care agent: part I. Stabilized hypochlorous acid: a component of the inorganic armamentarium of innate immunity. *Journal of burns and wounds* **6** (2007). PMC1853323 17492050

[49] McKenna SM, Davies K (1988). The inhibition of bacterial growth by hypochlorous acid. Possible role in the bactericidal activity of phagocytes. Biochemical Journal.

[50] Vissers MC, Winterbourn CC (1995). Oxidation of intracellular glutathione after exposure of human red blood cells to hypochlorous acid. Biochemical Journal.

[51] Kalyanaraman B, Sohnle PG (1985). Generation of free radical intermediates from foreign compounds by neutrophil-derived oxidants. The Journal of clinical investigation.

[52] Sakarya S, Gunay N, Karakulak M, Ozturk B, Ertugrul B (2014). Hypochlorous Acid: an ideal wound care agent with powerful microbicidal, antibiofilm, and wound healing potency. Wounds: a compendium of clinical research and practice.

[53] Chen X, Stewart PS (2000). Biofilm removal caused by chemical treatments. Water research.

[54] Babrowski T (2012). Pseudomonas aeruginosa virulence expression is directly activated by morphine and is capable of causing lethal gut derived sepsis in mice during chronic morphine administration. Annals of Surgery.

[55] Hirt H, Gorr SU (2013). Antimicrobial peptide GL13K is effective in reducing biofilms of Pseudomonas aeruginosa. Antimicrobial agents and chemotherapy.

[56] Dusane DH (2017). Effects of loading concentration, blood and synovial fluid on antibiotic release and anti-biofilm activity of bone cement beads. J Control Release.

[57] Fuest M, Boone C, Rangharajan KK, Conlisk AT, Prakash S (2015). A three-state nanofluidic field effect switch. Nano letters.

[58] Fuest M, Rangharajan KK, Boone C, Conlisk A, Prakash S (2017). Cation Dependent Surface Charge Regulation in Gated Nanofluidic Devices. Analytical Chemistry.

[59] Kellie BM, Silleck AC, Bellman K, Snodgrass R, Prakash S (2013). Deposition of few-layered graphene in a microcombustor on copper and nickel substrates. RSC Advances.

[60] Das A, Ganesh K, Khanna S, Sen CK, Roy S (2014). Engulfment of apoptotic cells by macrophages: a role of microRNA-21 in the resolution of wound inflammation. J Immunol.

[61] Roy S, Khanna S, Nallu K, Hunt TK, Sen CK (2006). Dermal wound healing is subject to redox control. Molecular therapy: the journal of the American Society of Gene Therapy.

[62] Das A (2016). Correction of MFG-E8 Resolves Inflammation and Promotes Cutaneous Wound Healing in Diabetes. J Immunol.

