# Supplementary material for: Electroceutical Treatment of Pseudomonas aeruginosa Biofilms
Source: Sci Rep. 2019 Feb 14;9:2008. doi: 10.1038/s41598-018-37891-y (PMC6375951; doi:10.1038/s41598-018-37891-y)
Supplement: Supplementary file 1 — Supplementary information [file 41598_2018_37891_MOESM1_ESM.docx]

**Electroceutical Treatment of *Pseudomonas aeruginosa* Biofilms**

Devendra H. Dusane ^a,^ *, Varun Lochab ^b,^*, Travis Jones ^b,^*, Casey W. Peters ^a^, Devin Sindeldecker ^a^, Amitava Das ^c,d^, Sashwati Roy ^c,d^, Chandan K. Sen ^c,d^, Vish V. Subramaniam ^b^, Daniel J. Wozniak ^a^, Shaurya Prakash ^b, †^, Paul Stoodley ^a,e, †^

^a^ Department of Microbial Infection and Immunity, The Ohio State University, Columbus, Ohio 43210.

^b^ Department of Mechanical and Aerospace Engineering, The Ohio State University, Columbus, Ohio 43210.

^c^ Department of Surgery, IU Health Comprehensive Wound Center, Indiana Center for Regenerative Medicine and Engineering, Indiana University School of Medicine, Indianapolis, IN 46202.

^d^ Comprehensive Wound Center and Department of Surgery, The Ohio State University Wexner Medical Center, Columbus, Ohio 43210.

^e^ Department of Orthopaedics; The Ohio State University, Columbus, Ohio 43210.

^†^Corresponding authors *e-mail address:* Paul.Stoodley@osumc.edu, prakash.31@osu.edu

*equal contribution

**Supplementary Discussion Data**

***Pseudomonas aeruginosa* lawns on agar surfaces are in biofilm mode of growth**

Bacterial cultures grown as lawns and colonies show characteristics of a biofilm, specifically, biofilms are known to harbor complex structural and biological attributes, such as the presence of an extracellular polymeric matrix, physical and chemical heterogeneity, and drug tolerance ^1,2^. These structural and biological attributes provide remarkable therapeutic challenges, including antimicrobial resistance.

Furthermore, bacterial colonies formed on solid growth media reproduce many properties of a biofilm, particularly high cell density and gradients (e.g., gradients in gas, nutrient, and metabolites)^3,4^. The highest reproducibility in such models is obtained by inoculating them as confluent bacterial lawns rather than separated colonies since colonies vary in size, and thus nutrient availability and differential gaseous gradients within each colony will increase variance^5,6^. More recently, agar colonies were used in Bacillus biofilm formation where complex colonial morphologies have been used as indicators of variation in biofilm phenotype ^7-9^. Bacterial growth on the surface of an agar plate can be distinguished from growth on hard surfaces since the biofilm is fed from the substratum. A similar situation may occur in biofilms associated with soft tissue infections^10^ such as those associated with cystic fibrosis^11^. Moreover, cells sampled from agar plate surfaces have exhibited markedly lower susceptibility towards drugs^12^ and host components^13^ when compared to the same cells grown planktonically in broth cultures. In other words, the structural, chemical, and functional attributes of bacterial lawns show significant similarities to biofilms with literature extending over 3 decades. Therefore, in this context, use of a bacterial lawn should be acceptable if our lawns demonstrate similar attributes.

Notably, we share similar observations showing tolerance of *P. aeruginosa* (PA) Xen41 lawns towards the antibiotic tobramycin as compared to the planktonic cells (Fig. 1). As described for Fig. S1 in the revised manuscript, in order to determine the antibiotic susceptibility of planktonic and lawn culture of PA Xen41 against tobramycin, minimal bactericidal concentration (MBC) was analyzed. The MBC value for tobramycin was 4µg/mL against planktonic culture whereas no effect was evident when the culture was grown as lawns at concentrations of tobramycin as high as 16µg/mL. The data in Fig. S1 suggests that PA Xen41 when grown as lawns in our study exhibits a biofilm phenotype.

**Consideration of mass transport conditions in wounds**

The time taken for an antimicrobial agent to spread through tissue or body fluid to achieve therapeutic concentrations is an important factor in considering *in vitro* models. There are a number of factors associated with this transport which include the hydrodynamic conditions of the system and binding and reaction interactions between the agent and the medium it is moving through ^14^. In a pig, full-thickness burn model, biofilm aggregates of PA and *Acinetobacter baumannii* were observed near the surface of the wound but also at the base of the wound bed, embedded at an approximate 200 µm depth in the wound ^15^. Clinical specimens also demonstrate that biofilms can be deep in the wound bed ^16^. In fact the thickness of the biofilm aggregates (approximately 20 µm) was considerably less than the thickness of the wound, therefore mass transport within the wound environment is an important factor in determining delivery of an antimicrobial agent to the biofilm deeper in the wound.

Surprisingly, there is little known regarding mass transfer conditions of a weeping wound (no active flow, but slow oozing of the wound fluids). In order to estimate the mass transport characteristics, it may be useful to consider the Peclet number (Pe) of a wound bed. Pe is a non-dimensional parameter which compares the relative contribution of advection (flow with velocity u) to diffusion (for species of diffusivity, D_e_) with respect to mass transport for a characteristic length scale (L_c_) over which an agent is transported ^17^;

Pe = uL_c_/D_e_ [1]

Therefore, to obtain estimates, first we consider u. We assume a wound exudates 10 cc per day which is considered high using the Mulder classification ^18^, providing an upper limit for flow velocities for a wound. For a 1 x 1” (2.54 cm × 2.54 cm = 6.45 cm^2^) wound that is typically used in the pig model studies reported, this would represent an average u of 1.93 × 10^-7^ m/s. Next we consider L_c_. Again, using the pig model we use a characteristic length of 200 µm (2 × 10^-4^ m), reflecting the depth of the wound. Next we consider the D_e_. If we assume that the active agent produced by electrolysis at the anode is indeed HOCl, the bulk, binary diffusivity of HOCl in water at 37°C is approximately 2 × 10^-9^ m^2^/s ^19^. However, since the absolute viscosity (υ) of wound exudate (approximately 1 × 10^-2^ Pa.s for pus) ^20^ is approximately a factor of 10 higher than that of 0.9% saline (7.1 × 10^-4^ Pa.s) the D_e_ is expected to be less, and so the value of bulk D_e_ used here likely overestimates the diffusivity. Exudate also has a density (ρ) higher than 1020 kg/m^3^ due to a high protein concentration ^21^. The Pe is 0.019, which predicts a diffusion dominated (i.e., transport is limited by diffusion of a species) system. Thus, we believe that the use of a solid agar plate to assess the killing of biofilms by the electrochemical production of antimicrobial agents in a diffusion dominated system is a valid model for a first approximation of a biofilm infected wound environment *in vitro* model.

**SUPPLEMENTARY REFERENCES**

1 Stoodley, P., Sauer, K., Davies, D. G. & Costerton, J. W. Biofilms as complex differentiated communities. *Annu Rev Microbiol* **56**, 187-209, doi:10.1146/annurev.micro.56.012302.160705 (2002).

2 Koo, H., Allan, R. N., Howlin, R. P., Stoodley, P. & Hall-Stoodley, L. Targeting microbial biofilms: current and prospective therapeutic strategies. *Nature reviews. Microbiology* (2017).

3 Stewart, P. S. Diffusion in Biofilms. *Journal of Bacteriology* **185**, 1485-1491, doi:10.1128/JB.185.5.1485-1491.2003 (2003).

4 Tuson, H. H. & Weibel, D. B. Bacteria-surface interactions. *Soft matter* **9**, 4368-4380, doi:10.1039/C3SM27705D (2013).

5 Hodgson, A. E., Nelson, S. M., Brown, M. R. & Gilbert, P. A simple in vitro model for growth control of bacterial biofilms. *The Journal of applied bacteriology* **79**, 87-93 (1995).

6 Gilbert, P., Allison, D. G., Evans, D. J., Handley, P. S. & Brown, M. R. Growth rate control of adherent bacterial populations. *Applied and environmental microbiology* **55**, 1308-1311 (1989).

7 Kearns, D. B. & Losick, R. Cell population heterogeneity during growth of Bacillus subtilis. *Genes & Development* **19**, 3083-3094, doi:10.1101/gad.1373905 (2005).

8 Verhamme, D. T., Murray, E. J. & Stanley-Wall, N. R. DegU and Spo0A jointly control transcription of two loci required for complex colony development by Bacillus subtilis. *J Bacteriol* **191**, 100-108, doi:10.1128/jb.01236-08 (2009).

9 Murray, E. J., Strauch, M. A. & Stanley-Wall, N. R. SigmaX is involved in controlling Bacillus subtilis biofilm architecture through the AbrB homologue Abh. *J Bacteriol* **191**, 6822-6832, doi:10.1128/jb.00618-09 (2009).

10 Wolcott, R. D., Kennedy, J. P. & Dowd, S. E. Regular debridement is the main tool for maintaining a healthy wound bed in most chronic wounds. *Journal of wound care* **18**, 54-56, doi:10.12968/jowc.2009.18.2.38743 (2009).

11 Costerton, J. W., Lam, J., Lam, K. & Chan, R. The role of the microcolony mode of growth in the pathogenesis of Pseudomonas aeruginosa infections. *Reviews of infectious diseases* **5 Suppl 5**, S867-873 (1983).

12 Al-Hiti, M. M. & Gilbert, P. A note on inoculum reproducibility: a comparison between solid and liquid culture. *The Journal of applied bacteriology* **55**, 173-175 (1983).

13 DeMatteo, C. S. *et al.* Susceptibility of Pseudomonas aeruginosa to serum bactericidal activity. A comparison of three methods with clinical correlations. *The Journal of laboratory and clinical medicine* **98**, 511-518 (1981).

14 Stewart, P. S. & Raquepas, J. B. Implications of reaction-diffusion theory for the disinfection of microbial biofilms by reactive antimicrobial agents. *Chemical engineering science* **50**, 3099-3104 (1995).

15 Roy, S. *et al.* Mixed‐species biofilm compromises wound healing by disrupting epidermal barrier function. *The Journal of pathology* **233**, 331-343 (2014).

16 Barker, J. C., Khansa, I. & Gordillo, G. M. A Formidable Foe Is Sabotaging Your Results: What You Should Know about Biofilms and Wound Healing. *Plastic and reconstructive surgery* **139**, 1184e-1194e (2017).

17 Stewart, P. S. Mini-review: convection around biofilms. *Biofouling* **28**, 187-198 (2012).

18 Mulder, G. D. Quantifying wound fluids for the clinician and researcher. *Ostomy/wound management* **40**, 66-69 (1994).

19 Chao, M. The diffusion coefficients of hypochlorite, hypochlorous acid, and chlorine in aqueous media by chronopotentiometry. *Journal of The Electrochemical Society* **115**, 1172-1174 (1968).

20 Simpson, G., Roomes, D. & Heron, M. Effects of streptokinase and deoxyribonuclease on viscosity of human surgical and empyema pus. *CHEST Journal* **117**, 1728-1733 (2000).

21 Cutting, K. F. Wound exudate: composition and functions. *British journal of community nursing* **8**, S4-S9 (2003).

**SUPPLEMENTARY FIGURES**


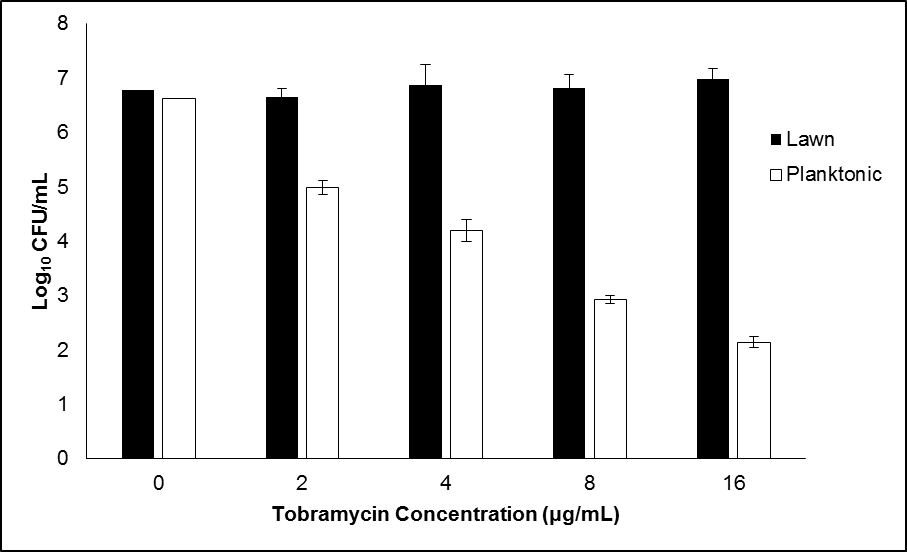


**Supplementary Figure S1:** Minimum bactericidal concentration (MBC) of tobramycin against planktonic and lawn cultures of *P. aeruginosa* Xen41. Note the nearly 4 orders of magnitude reduction in the planktonic form, whereas the lawn is hardly affected. This antibiotic resistance is one of the hallmarks of a bacterial biofilm and used here to illustrate that our bacterial lawn is indeed a biofilm.


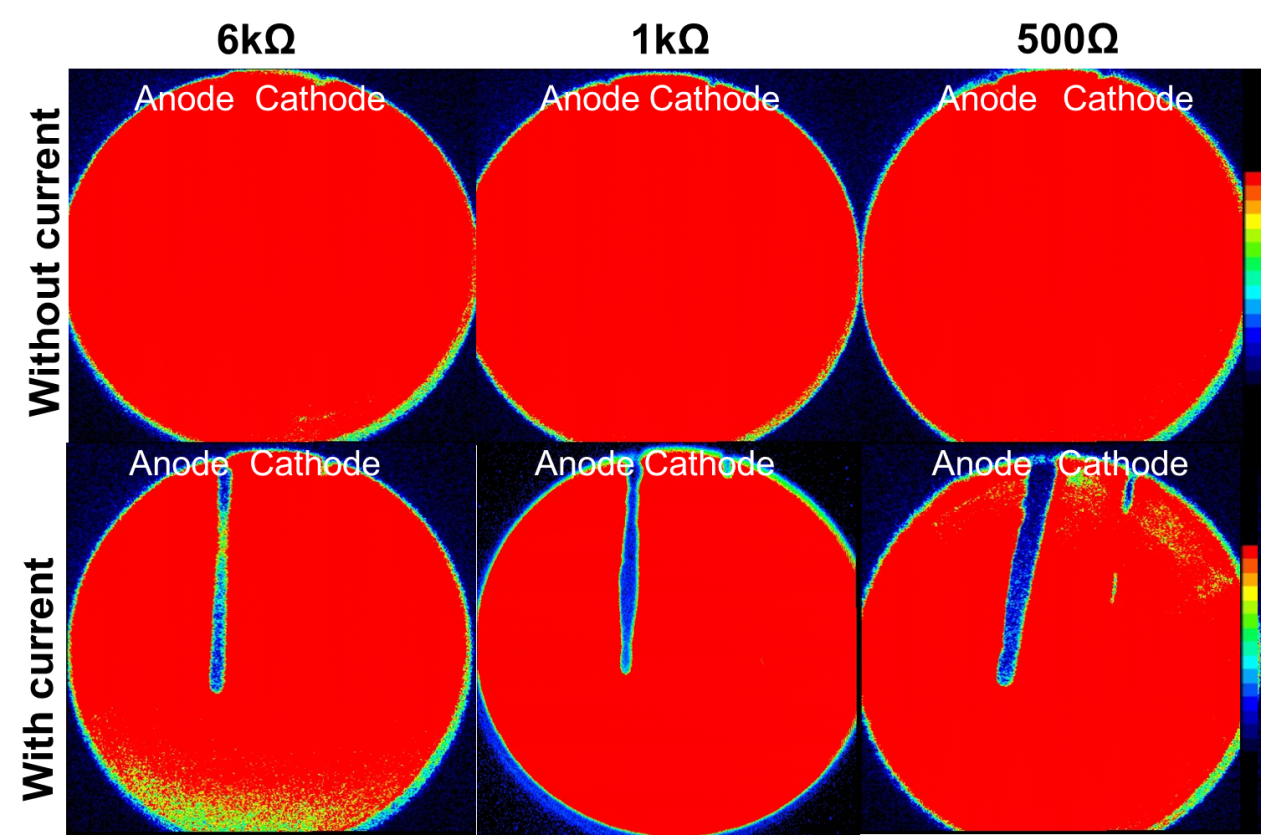


**Supplementary Figure S2:** IVIS images showing effect of varying ballast resistor on killing of lawn biofilms of 24 h grown *P. aeruginosa* Xen41. There appeared to be a “dose response” with reducing ballast resistance, however with the 500Ω ballast, there appeared to be physical changes in the agar (not apparent in the IVIS image) suggesting heating may be an issue at the higher levels of electrical current.


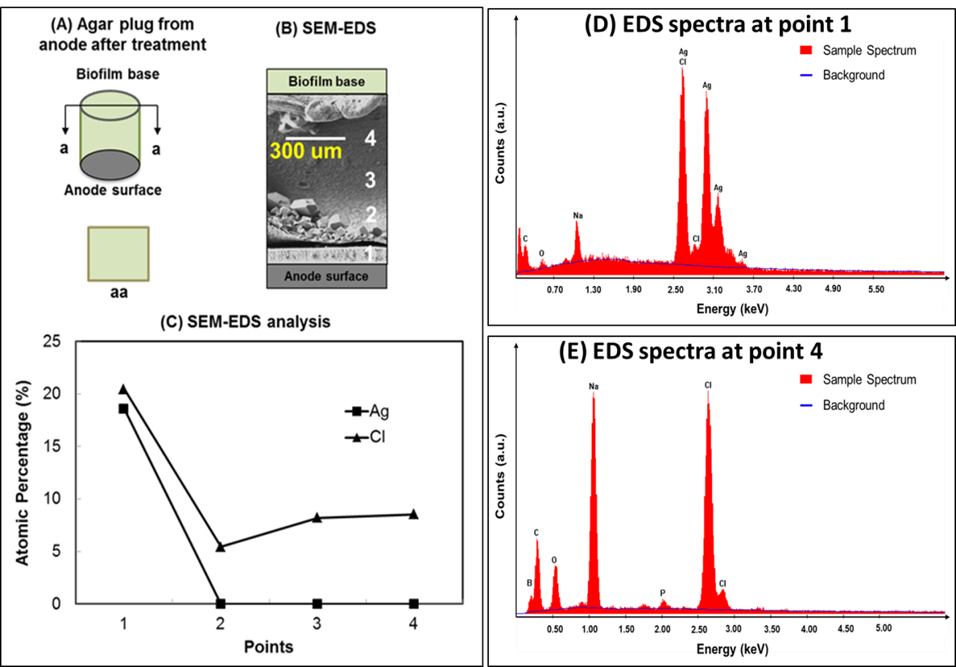


**Supplementary Figure S3:** Energy dispersive spectroscopy (EDS) of *P. aeruginosa* lawn biofilm agar plug from anode after applying current for 24 h, A) 6 mm diameter agar plug with cross section (aa). B) SEM imaging and EDS measured at four different points from the anode surface to the base of the biofilm. C) Relative atomic percentage of Ag and Cl at points 1-4. D) EDS spectrum at point 1 showing presence of Ag and E) EDS spectrum at point 4 showing absence of Ag in the medium (i.e., indicating no leaching of Ag).


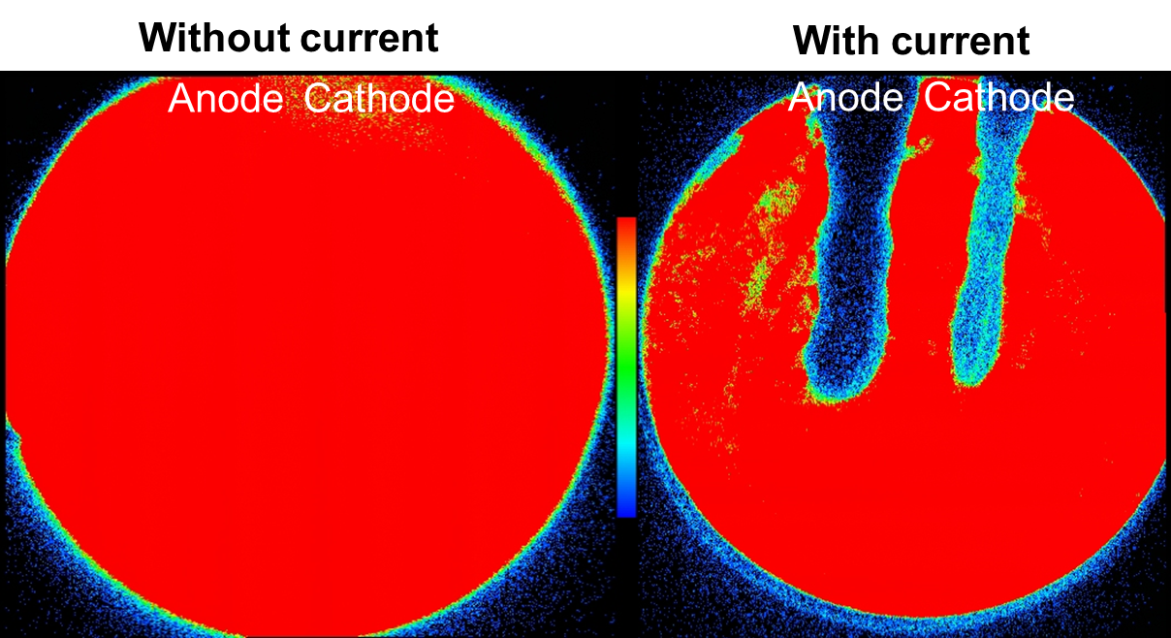


**Supplementary Figure S4:** Killing of lawn biofilms of *P. aeruginosa* at both the anode and cathode after applying current via gold (Au) electrodes.


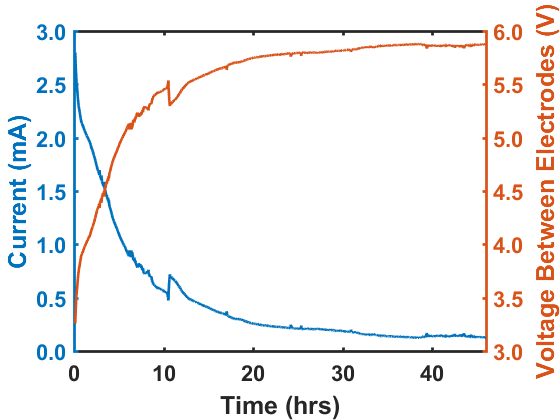


**Supplementary Figure S5:** Current measured between silver electrodes as a function of time using 6 V battery (2 batteries of 3V in series) with a 1 kΩ ballast current limiter.

The measured current and voltage drop across the two electrodes and through the agar and any adjoining electrode surface layers are shown here versus time, for a typical experiment. The voltage plotted here is $V=(6-1000I)$, where V is in volts and I is in Amperes. Note that under open circuit conditions, I=0, and V=6 Volts, which is the battery source. The instant current begins to flow, the resistance of the agar affects it and the current drops quickly to about 3.5 mA and continues to decline as electrochemical reactions at the electrode surfaces and in the agar change its resistance. Correspondingly, the voltage increases monotonically as the current decreases. Note also the sharp initial decrease in the current and steep increase in the voltage as the AgCl layer with its relatively high resistance, forms at the anode.
